# Change in Main Histological Type of Invasive Breast Cancer From Ductal to Lobular Carcinoma by Neoadjuvant Chemotherapy

**DOI:** 10.7759/cureus.43816

**Published:** 2023-08-20

**Authors:** Takako Okubo, Yoshimitsu Minari, Yoshihiro Ikura

**Affiliations:** 1 Pathology, Takatsuki General Hospital, Takatsuki, JPN; 2 Breast Surgery, Takatsuki General Hospital, Takatsuki, JPN

**Keywords:** mixed-type, substitution, neoadjuvant chemotherapy, invasive lobular carcinoma, invasive ductal carcinoma, histological type, breast cancer

## Abstract

We present a case study of breast cancer initially diagnosed as invasive ductal carcinoma (IDC), which subsequently substituted into invasive lobular carcinoma (ILC) following neoadjuvant chemotherapy (NAC). A 61-year-old woman presented with a palpable breast lump, and histological examination through core needle biopsy (CNB) confirmed the presence of IDC. After a 6-month course of NAC, the patient achieved a clinically complete response (cCR) and underwent mastectomy. The surgical specimen showed no detectable tumor upon palpation, but microscopic analysis revealed a highly infiltrative growth of poorly-cohesive small atypical cells in the original tumor area. Immunohistochemical staining demonstrated that the tumor cells were negative for E-cadherin, leading to a diagnosis of ILC. To address the histological discrepancy before and after NAC, we re-evaluated the initial CNB using E-cadherin immunohistochemistry. While most tumor cells were E-cadherin positive, a small area displaying scirrhous subtype-like morphology exhibited E-cadherin negativity. Consequently, we revised the diagnosis to mixed IDC-ILC. The differential chemosensitivity between IDC and ILC may provide insight into this phenomenon.

## Introduction

Neoadjuvant chemotherapy (NAC) is a systemic treatment administered before surgery for patients with cancers. As for breast cancers, it is typically recommended for patients with tumors larger than 2 cm and/or axillary lymph node metastases [1]. The primary goals of NAC are to reduce tumor size and enable breast-conserving surgery. Additionally, NAC provides an opportunity to assess the chemosensitivity of tumors through histological examination of resected samples [2,3]. Moreover, NAC can sometimes result in histological type alterations, which may impact patients' prognoses [4]. Chemotherapy-related cytological changes include nuclear pleomorphism, multinucleation, enhanced cytoplasmic transparency, vacuolization, and often attenuated cell-cell adhesion [5]. Notably, limited research has been conducted on the distinct changes in histological type before and after chemotherapy [4,6].

In this report, we present a case of breast cancer where the initial diagnosis based on core needle biopsy (CNB) indicated invasive ductal carcinoma (IDC). However, the histological examination of the surgical specimen after NAC revealed invasive lobular carcinoma (ILC).

## Case presentation

**Figure 1:**
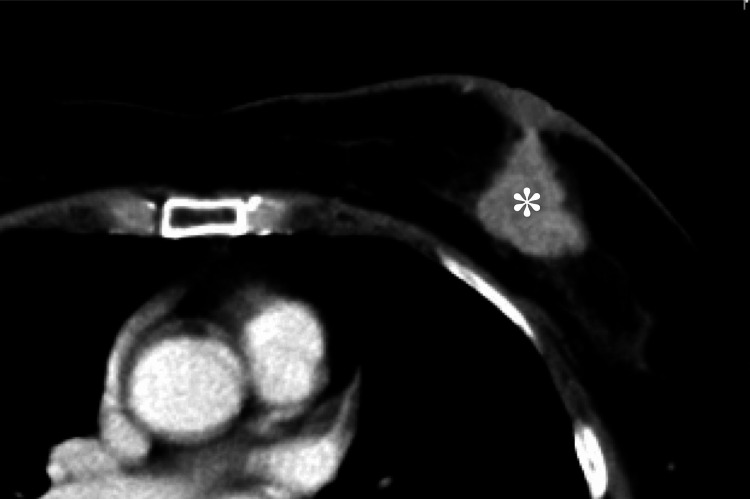
A chest CT image prior to NAC An irregular nodule is seen in the left breast (asterisk), indicative of breast cancer. Intraductal spreading towards the nipple is suspected. NAC: Neoadjuvant chemotherapy

A 61-year-old woman presented to our hospital with a firm breast lump near the left nipple. Upon examination by a breast surgeon, a large mass (approximately 5 cm) was detected in the DE region. Mammography revealed an indistinct mass with microcalcification in the middle outer left breast. Ultrasonography, CT, and MRI confirmed the presence of a 46x39x23 mm tumor in the same area, extending to the nipple via intraductal spreading (Figure 1). Enlarged left axillary lymph nodes indicated possible metastasis, while no metastatic foci were observed in the lungs or liver. Positron emission tomography CT was not performed due to its limited necessity in this case.

**Figure 2:**
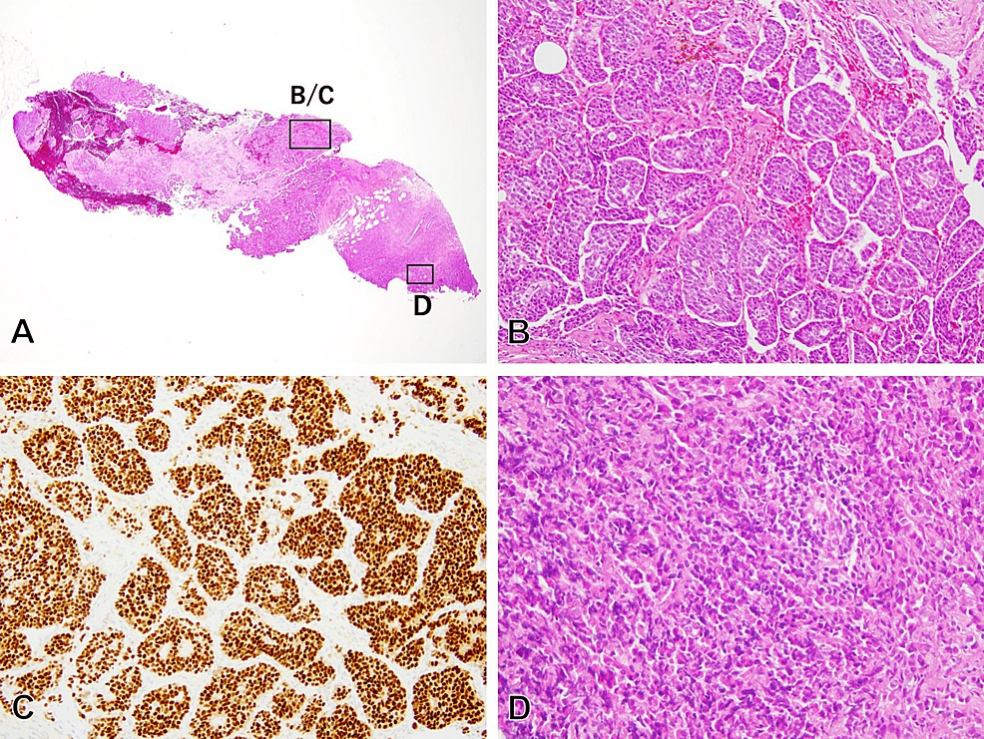
Histological examination of the pre-NAC CNB (A) Low-magnification image showing irregular cell aggregation and fibrosis (HE, original magnification, x20); (B) The proliferative lesion primarily consists of atypical polygonal cells exhibiting cohesive growth, forming multiple cellular nests, and occasional glandular structures (HE, original magnification, x200); (C) Most tumor cells strongly express ER (Immunoperoxidase with anti-ER, original magnification, x200); (D) Scirrhous-like area accompanied by inflammatory infiltration is observed at the periphery of the sample (HE, original magnification, x400) NAC: Neoadjuvant chemotherapy; CNB: Core needle biopsy; HE: Hematoxylin-eosin; ER: Estrogen receptor

A CNB was performed for histopathologic diagnosis. Hematoxylin-eosin (HE) staining showed proliferation of atypical polygonal cells forming small nests with occasional glandular structures (Figure 2A-2C). Based on morphology, the tumor was initially diagnosed as IDC (histological grade 2), predominantly exhibiting a solid growth pattern with a small area of scirrhous pattern associated with mononuclear cell infiltration (Figure 2D). Immunohistochemical analysis revealed positive hormone receptor expression (estrogen receptor (ER) positive, progesterone receptor positive), negative human epidermal growth factor receptor 2 (HER2), and a Ki-67 labeling index of 30%. These findings categorized the tumor as Luminal B-like subtype. Fine needle aspiration of the axillary lymph node confirmed metastasis. According to the TNM classification, the patient was diagnosed with Stage IIB (cT2 N1 M0) breast cancer.

**Figure 3:**
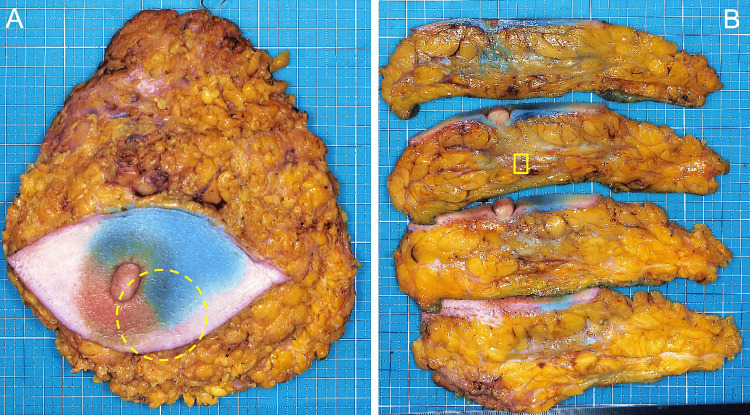
Macroscopic examination of the resected sample following NAC (A) No palpable tumor was detected. The approximate location of the original tumor is indicated by an open circle of yellow broken line; (B) Even upon observation of the cut surface, no tumor was identified. A region histologically shown as ILC in Figure 4 is indicated by an open square of yellow continuous line. NAC: Neoadjuvant chemotherapy; ILC: Invasive lobular carcinoma

**Figure 4:**
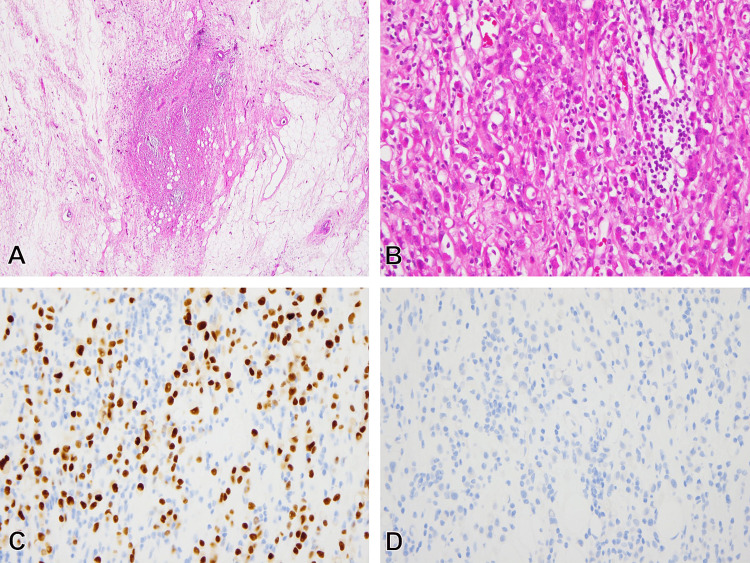
Histological evaluation of the surgical specimen after NAC (A) No residual is observed, but a peculiar fibrotic lesion is present (HE, original magnification, x40); (B) A high-magnification image of the fibrotic lesion reveal poorly cohesive, atypical small cells with a highly infiltrative growth pattern (HE, original magnification, x400); (C,D) Immunohistochemical findings indicate ER positivity (C) and E-cadherin negativity (D) in the cells (both immunoperoxidase method, original magnification, x400) NAC: Neoadjuvant chemotherapy; IDC: Invasive ductal carcinoma; HE: Hematoxylin-eosin; ER: Estrogen receptor

As per the standard treatment for Stage IIB breast cancer, the patient underwent NAC with dose-dense adriamycin + cyclophosphamide, and docetaxel for six months, followed by mastectomy [1]. Prior to surgery, no palpable tumor was present, indicating a clinically complete response (cCR) to NAC. In order to enhance the radicalness of the treatment, however, a total mastectomy along with axillary lymph node dissection was adopted as surgery. Macroscopically, no discernible tumor was observed in the resected breast sample, even upon detailed examination with 5-mm-thick slices (Figure 3). However, microscopic evaluation revealed highly infiltrative growth of poorly-cohesive atypical small cells in the same region as the original tumor (Figure 4A, 4B). These proliferating cells exhibited ER+ and cytokeratin+ expression but were E-cadherin- (Figure 4C, 4D). The lesion was diagnosed as ILC, suggesting substitution from IDC to ILC following NAC. Both the histological grade and the biomarker expressions of the ILC were consistent with those of the original IDC. No residual IDC was detected in the breast sample, and two of the 14 axillary lymph nodes were positive for metastases only of the ILC component.

**Figure 5:**
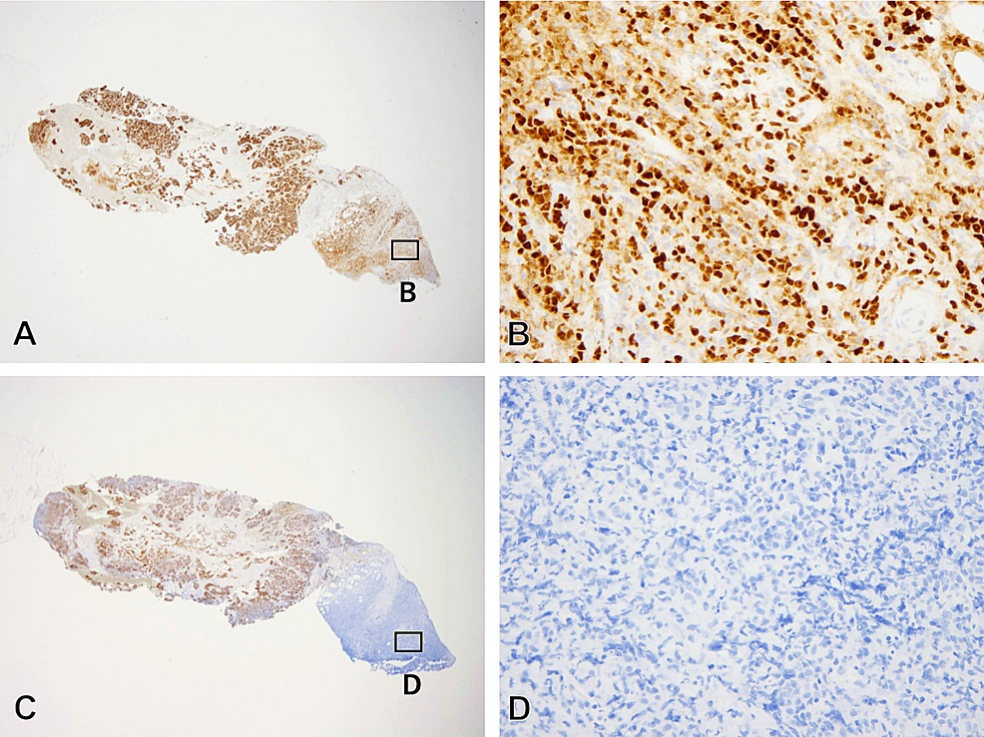
Re-examination findings of the initial CNB specimen (corresponding to Figure 2A and 2D) (A,B) The tumor cells exhibit ER positivity, including the scirrhous-like area (immunoperoxidase with anti-ER) (original magnifications, x20 (A) and x400 (B)); (C,D) The scirrhous-like area demonstrates E-cadherin negativity, while the tumor cells mostly display E-cadherin positivity (immunoperoxidase with anti-E-cadherin) (original magnifications, x20 (C) and x400 (D)) CNB: Core needle biopsy; ER: Estrogen receptor

We re-examined the CNB specimen using E-cadherin immunohistochemistry. While most tumor cells were E-cadherin+, a small portion of tumor cells seen morphologically as scirrhous-patterned IDC were E-cadherin- (Figure 5). Consequently, we revised the diagnosis of the original tumor to mixed IDC-ILC.

The postoperative course was uneventful, and the patient commenced adjuvant therapy consisting of an aromatase inhibitor and radiation (50 Gray). Currently, there is no evidence of tumor recurrence.

## Discussion

To date, several cases of histologic type substitution after NAC have been reported, with most cases involving changes from IDC to other types or mixed tumors [4,6]. In our case, the pretreatment CNB exhibited features consistent with typical IDC, but following six months of NAC, ILC (rather than IDC) was identified in the same area. However, upon a detailed review of the pre-NAC CNB specimen using E-cadherin immunohistochemistry, the small component initially interpreted as IDC scirrhous subtype was determined to be ILC. Thus, we concluded that only the IDC component of the mixed IDC-ILC responded to NAC, while the ILC component survived, grew, and expanded.

The difference in chemosensitivity between IDC and ILC has been discussed in previous studies, with most reports suggesting that ILC is less sensitive to chemotherapy [7-9]. Some propose that this tendency may be attributed to the rich stroma in ILC, while others suggest that intrinsic immunohistochemical and molecular factors, regardless of histological types, could influence the efficacy of chemotherapy [7-9]. In our case, the biomarker expressions were nearly identical in both the IDC and ILC components, including the residual tumor after NAC, all of which were categorized as the common intrinsic subtype (Luminal B-like). Their histological grades were also identical. These imply that factors other than histological grades or biomarkers/intrinsic-subtypes contribute to the differences in chemosensitivity between IDC and ILC.

Remarkably, there are few reports documenting pure ILC arising from mixed IDC-ILC after NAC, as in the present case. Troxell et al. reported a case of simultaneous heterotopic double cancers comprising IDC and ILC, where only the ILC component remained after chemotherapy [5]. The IDC and ILC components exhibited different intrinsic subtypes, namely triple-negative and Luminal A-like, respectively. Gahlaut et al. reported six cases (approximately 4%) that transformed from IDC to mixed IDC-ILC [4]. This suggests that the ILC components, which were not detected in the pre-NAC biopsy, may have manifested after NAC. Therefore, it is crucial for pathologists to be aware that NAC can leave behind a chemo-resistant component of a mixed tumor, which may impact treatment prognosis. When a pathologist identifies a tumor component that differs from the main histological type in a biopsy specimen, regardless of its small extent, it should not be disregarded as a minor component, but rather should be precisely reported as a mixed-type.

## Conclusions

We presented a case of mixed IDC-ILC in which the primary histologic type changed from IDC to ILC after NAC. The efficacy of NAC may vary depending on histological differences, even within the tumor components comprising a single instance of breast cancer. To enhance the prediction accuracy of treatment efficacy, it is essential to remain mindful that, in addition to the main histological type, minor components that are challenging to recognize in a biopsy specimen may be concealed by morphology modifiers, such as inflammation.

## References

[1] Pelizzari G, Gerratana L, Basile D (2019). Post-neoadjuvant strategies in breast cancer: from risk assessment to treatment escalation. Cancer Treat Rev.

[2] Kümmel S, Holtschmidt J, Loibl S (2014). Surgical treatment of primary breast cancer in the neoadjuvant setting. Br J Surg.

[3] Baker GM, King TA, Schnitt SJ (2019). Evaluation of breast and axillary lymph node specimens in breast cancer patients treated with neoadjuvant systemic therapy. Adv Anat Pathol.

[4] Gahlaut R, Bennett A, Fatayer H (2016). Effect of neoadjuvant chemotherapy on breast cancer phenotype, ER/PR and HER2 expression - implications for the practising oncologist. Eur J Cancer.

[5] Troxell ML, Gupta T (2022). Neoadjuvant therapy in breast cancer: histologic changes and clinical implications. Surg Pathol Clin.

[6] Sethi D, Sen R, Parshad S, Khetarpal S, Garg M, Sen J (2013). Histopathologic changes following neoadjuvant chemotherapy in locally advanced breast cancer. Indian J Cancer.

[7] Lips EH, Mukhtar RA, Yau C (2012). Lobular histology and response to neoadjuvant chemotherapy in invasive breast cancer. Breast Cancer Res Treat.

[8] Riba LA, Russell T, Alapati A, Davis RB, James TA (2019). Characterizing response to neoadjuvant chemotherapy in invasive lobular breast carcinoma. J Surg Res.

[9] Mathieu MC, Rouzier R, Llombart-Cussac A (2004). The poor responsiveness of infiltrating lobular breast carcinomas to neoadjuvant chemotherapy can be explained by their biological profile. Eur J Cancer.

